# Biomaterials for hollow organ tissue engineering

**DOI:** 10.1186/s13069-016-0040-6

**Published:** 2016-03-23

**Authors:** Eseelle K. Hendow, Pauline Guhmann, Bernice Wright, Panagiotis Sofokleous, Nina Parmar, Richard M. Day

**Affiliations:** Applied Biomedical Engineering Group, Division of Medicine, University College London, 21 University Street, London, UK

**Keywords:** Tissue engineering, Biomaterials, Regenerative medicine, Scaffold, Biocompatible

## Abstract

Tissue engineering is a rapidly advancing field that is likely to transform how medicine is practised in the near future. For hollow organs such as those found in the cardiovascular and respiratory systems or gastrointestinal tract, tissue engineering can provide replacement of the entire organ or provide restoration of function to specific regions. Larger tissue-engineered constructs often require biomaterial-based scaffold structures to provide support and structure for new tissue growth. Consideration must be given to the choice of material and manufacturing process to ensure the de novo tissue closely matches the mechanical and physiological properties of the native tissue. This review will discuss some of the approaches taken to date for fabricating hollow organ scaffolds and the selection of appropriate biomaterials.

## Background

### Introduction

The increasingly ageing population represents a huge socioeconomic burden on healthcare systems worldwide. New therapies are urgently sought that can rejuvenate or restore function of physiological systems that no longer function correctly due to the cumulative effects of wear and tear. Organ transplantation offers a limited solution due to donor shortages and the need for lifelong immunosuppression [1]. Xenotransplantation continues to be proposed but has yet to fulfil its promise due to concerns over long-term stability, immune system rejection and zoonoses [2]. Despite recent advances in genome editing for xenotransplantation, there are still many challenges to overcome before organ transplant from animals becomes a commonplace [3].

There is therefore a clear need for alternative strategies aimed at producing curative treatments. Tissue engineering is a rapidly developing interdisciplinary field that involves both materials science and engineering with medical research to address clinical problems. The majority of tissue engineering strategies rely on the use of biocompatible materials to facilitate tissue regeneration. Scaffolds can be manufactured from different materials and by a variety of methods, depending on the intended application. Three-dimensional scaffolds have the ability to guide tissue regrowth, provide support, encourage cell adhesion and proliferation, be combined with biological agents to provide a sustained release of various factors or drugs, and support neovascularisation within an interconnected porous network [4–6]. Tissue engineering can in theory be applied to any part of the human body, but advances in the bioengineering of hollow organs have led to several high-profile success stories in recent years, notably artificial bladders successfully transplanted into patients in 2006 [7] and a trachea implantation in 2008 [8, 9]. The objective of this review is to discuss different biomaterials and tissue engineering strategies for hollow organs found in the cardiovascular, respiratory and gastrointestinal systems.

### Scaffold fabrication techniques

There are many different methods available to fabricate scaffolds for tissue engineering. The approach taken is often determined by the starting material, scale of the construct, and physiochemical and mechanical properties of the scaffold. Techniques that have been used to produce scaffolds suitable for hollow organ tissue engineering include electrospinning and extrusion methods that allow for the production of polymer fibres or meshes [10–12], thermally induced phase separation (TIPS) [11], electrohydrodynamic (EHD) processing [13], 3D printing [14] and hydrogels, whose properties can be controlled by changes on pH or temperature [15].

For a material to be classed as a biomaterial, it must fulfil certain criteria. The material should be compatible with cells and tissues in the local milieu so as not to illicit a chronic inflammatory response. Other desirable properties may include biodegradation, porosity, specific mechanical properties and the ability to facilitate cell attachment [16, 17]. Synthetic polymers are often chosen because their composition and structure can be refined to meet these specifications. They are often cheaper to produce than natural polymers, and greater control can be exerted over the manufacturing processes, which provides scope for reproducible and scaled-up manufacture. A widely used class of synthetic polymer is poly(alpha-hydroxyesters) due to their capacity to offer control of degradation and porosity which can affect the behaviour of cells [18–20]. A variety of natural polymers have also been investigated for use as scaffold materials, such as collagen and elastin found in the extracellular matrix of many tissues. These materials are generally considered to offer better cell-material interactions compared with synthetic materials since they provide an environment that resembles native tissue. However, these materials are often more difficult to manipulate and process into scaffold structures compared with synthetic materials [10]. The advantages and disadvantages of some of the different scaffold fabrication techniques available are outlined in Table 1.

**Table 1:** Various fabrication techniques for biomaterial scaffolds

Fabrication method	Application	Advantages	Disadvantages	References
Tissue decellularisation	Tissues with high ECM content, e.g. trachea, heart valves	Native composition (ECM), retains mechanical properties and shape of organ	Immunogenicity due to incomplete decellularisation, loss of ECM, requires donor organ	[5, 10, 78]
Electrohydrodynamic (EHD) processing	Drug delivery, hard and soft tissue engineering, wound healing	Fibres, particles and encapsulated particle production, biocompatible, biodegradable, manufacturing parameters adjustable to tailor product, control over pore size and distribution	Inhomogeneous distribution of seeded cells	[5, 10, 13, 79, 80]
Electrospinning	Drug delivery, hard and soft tissue engineering, wound healing	Production of fibres and encapsulated fibres, high porosity, surface area, biocompatible and biodegradable, manufacturing parameters adjustable to tailor product	Inhomogeneous distribution of seeded cells	[81, 82]
Hydrogels	Scaffolds for cartilage, connective tissue and soft tissue bioengineering, cell delivery, drug delivery, wound healing	Tuneable biodegradability, biomimicry, biocompatible, improves cellular interactions, mimics native ECM, injectable, self-assembly possible in response to pH and temperature, can be incorporated with other materials	Limited mechanical properties, sensitive to the surrounding environment	[5, 10, 15, 28–31]
Thermally induced phase separation (TIPS)	Microparticles for tissue engineering, cell delivery, drug delivery	High porosity, biocompatible, biodegradable, 3D scaffold, manufacturing parameters adjustable to tailor product, interconnected porous network, cell proliferation, injectable	Limited open space through scaffold, inhomogeneous size particles, particle aggregation	[10, 11, 80]
3D printing	Fully developed constructs	Complex structures mechanically similar to native tissue, fast processing	Limited materials, post-processing	[4, 14, 27]

### Bioengineered blood vessels

Cardiovascular diseases are within the top ten causes of death worldwide, with pathologies including myocardial infarction, stroke, congestive heart failure, peripheral arterial disease and vascular disease [21]. The need for curative therapies is clear, and the use of biomaterial scaffolds alongside cell therapies could provide this [22]. Biomaterials can be utilised in a variety of ways from the use of microparticles to successfully deliver cells to their target site [23] to incorporating growth factors or drugs in a degradable material for their sustained release.

One of the most popular cell types studied in bioengineering blood vessels is endothelial cells, which line arteries and veins and form capillaries. They control the function of the entire vessel by signalling to the surrounding smooth muscle tissue to response to changes in shear stress by adapting lumen diameter and wall thickness [24]. Other cell types within blood vessels such as smooth muscle cells and pericytes are also researched to promote angiogenesis [25] and bioengineer blood vessels [26].

Whole blood vessels can be produced by various tissue engineering methods including 3D printing [27], 3D patterns on hydrogels [28] and composite scaffolds [29–31]. Recent studies into whole vessel engineering have focused on the use of polymer scaffolds incorporated with hydrogels. Hydrogels are defined as three-dimensional hydrophilic cross-linked networks that have the ability to absorb large amounts of water. Hydrogels are being increasingly considered as a material of use in tissue engineering applications due to their ability to mimic natural tissues and support tissues [32]. Singh et al. (2013) constructed a polyethylene glycol (PEG)-collagen composite scaffold containing photoencapsulated endothelial cells and fibroblasts to produce vessel networks within well-defined hollow lumens [30]. In addition, a novel method using 3D printing to create patterns on polycaprolactone (PCL) surfaces coated with gelatin allowed smooth muscle cells to align to the patterns and so facilitate blood vessel formation.

New blood vessels have the ability to grow from the existing vessels throughout adult life in a process called angiogenesis. Angiogenesis is dependent on growth factors such as vascular endothelial growth factor (VEGF) and basic fibroblast growth factor (bFGF), as well as signalling and interactions between cell types such as endothelial cells and pericytes [33]. Promotion of angiogenesis through the use of biomaterials, cell therapies and administration of growth factors is widely researched.

Bauters et al. (1995) showed that administering VEGF into a rabbit model of hind limb ischemia resulted in improved blood vessel formation compared to a control group after 30 days [34]. Unfortunately, VEGF and other growth factors have short half-lives, some only minutes long [35]. Therefore, once an injection is administered, VEGF begins to degrade. To overcome this, larger doses or more frequent injections could be given. Excessive VEGF levels can, however, have oncogenic effects [36]. Another option is to encapsulate VEGF in a biodegradable material so it can be slowly released as the material degrades. Rocha et al. (2008) encapsulated VEGF in poly(lactic-co-glycolic acid) (PLGA) microspheres and implanted them in a tissue-engineered intestine in order to promote epithelialisation. In vitro and in vivo studies showed that the microspheres had a sustained release of active VEGF, and in vivo studies also found that compared to the controls, the group given the VEGF particles had increased capillary density, epithelial cell proliferation and larger intestinal constructs [37]. Cell therapy can also be used to promote angiogenesis by encouraging vessel formation by implanting stem cells or cells found in blood vessels to initiate angiogenesis by producing growth factors. Chen et al. (2005) implanted bone marrow stromal cells into the ischemic boundary zone after rats had a stroke. Results also showed that the rats implanted with cells have significantly higher VEGF levels which accounts for the enlarged blood vessels and newly formed capillaries in comparison to rats treated with phosphate buffered saline (PBS) [38]. Numerous studies have shown that simply injecting cells into ischemic tissue can result in a large cell loss from the target site. Hong et al. (2013) showed that 1 day after stem cells were injected into heart infarcts, only 10 % of cells remained at the injection site [23]. An alternative solution is to utilise cell-based biomaterial therapies, specifically the use of microspheres, to allow adhesion to the implant site and allow cells to attach and proliferate, increasing the residence time of cells and consequently enhancing their therapeutic effect. Promoting the regeneration of blood vessels through angiogenesis or engineering entire constructs can treat a variety of cardiovascular diseases as well as being vital for the survival of engineered tissues by allowing vascularisation.

### Gastrointestinal tissue engineering

The gastrointestinal (GI) tract consists of the oesophagus, stomach, small and large intestines and colon. It is a complex hollow organ system with diverse functions and structures that facilitate digestion, absorption of nutrients and excretion of waste from the body. Engineered GI tissue can be applied to repair damage caused by stomach cancer, inflamatory bowel disease, as well as replace sphincter tissue to cure faecal and urinary incontinence.

Stomach cancer is the fourth most common fatal cancer in Europe [39]. Gastrectomy is the recommended treatment for stomach cancer (alongside chemotherapy and radiotherapy if the cancer has metastasised) [40] but has adverse side effects such as malnutrition, reflux esophagitis, osteomalacia and anaemia. The alternative is to remove just the cancerous tissue in order to preserve the stomach; however, as a result of this, the stomach often becomes distorted and unable to function fully. The stomach is notoriously difficult to engineer due to its shape and scale, and as a result, relatively few studies have addressed this need [40]. Rather than attempting to directly replicate the native stomach, more attention has been placed on producing tubular constructs and patches. Othman et al. (2015) cultured cells on collagen and polyethylene terephthalate (PET) sheets that were rolled into 3D scaffolds by an automated tube fabricator. By using this method, they were able to produce large constructs with appropriate thickness, length and luminal diameter for gastrointestinal tissue engineering applications [41]. To repair patches of tissue after resection, typically a biodegradable material is used. There have been a few studies that have used polyglycolic acid (PGA) and poly-l-lactic acid (PLLA) seeded with endothelial cells from the stomach. Maemura et al. (2008) implanted these constructs in an in vivo rat model. They found that the cells on the tissue-engineered scaffold had comparable secretory functions to a native stomach [42]. The same group also demonstrated that there was less deformity and a smaller volume loss when the tissue-engineered construct was implanted into a gastrointestinal deformity compared to controls [43]. Another group, Sala et al. (2009), successfully implanted a tissue-engineered stomach and small intestine (PGA and PLLA seeded with autologous stomach-derived organoid units) into an in vivo swine model. Histological analysis showed all intestinal cell types present after 7 weeks on the scaffold as well as mucus cells present in the stomach. These pre-clinical studies show that it is possible to generate a tissue-engineered construct at a large scale with the correct architecture, ability to facilitate cell attachment and proliferation, suggesting that it could be possible in humans requiring gastrointestinal transplants [44]. Unfortunately, to date, all of these studies have remained at the pre-clinical stage of development.

Inflammatory bowel disease (IBD) affects 1 in 250 people in the UK with the most prevalent examples being ulcerative colitis (UC) and Crohn’s disease. Both diseases are lifelong, with Crohn’s primarily affecting the small intestine and UC the large intestine [45]. In the UK, 20 % of people with UC and 60 % with Crohn’s will have to have surgery, and often, portions of the GI system are removed [46]. These surgeries are highly invasive and can result in stoma complications, short bowel syndrome and malnutrition [47]. Intestinal tissue engineering predominately focuses on the small intestine. Numerous in vivo studies have focused on large animal models using collagen sponge scaffolds seeded with intestinal smooth muscle to repair patches of intestinal tissue. Despite limited success with formation of epithelial layers, these studies have found it difficult to replicate the alignment and contractile function of smooth muscle cells in vivo which is vital for sufficient nutrient absorption [48–50]. An alternative cell source such as intestinal organoid units have also been used alongside biomaterials for small intestinal tissue engineering. Gritscheit et al.(2004) successfully lengthening short bowel defects in canine models of short bowel syndrome [51, 52]. Similarly for the small intestine, the large intestine requires the formation of longitudinal and circular smooth muscle layers to allow for peristalsis. Bitar et al. (2013) co-cultured smooth muscle cells with intestinal organoid units to produce circular constructs that were wrapped around tubular collagen scaffolds to resemble the native colon. Mechanical and physiological studies showed successful contractility in the construct, which is a promising step to engineer a functional intestine. Further in vivo studies are required to assess whether or not peristalsis can be replicated [53, 54].

Faecal incontinence is a major public health issue with no curative treatment available [55] with 10 % of people in the UK under 40 suffering from faecal and urinary incontinence [56]. This figure increases to 24 % in people over 40 years old [57, 58]. The most common cause of incontinence in women is obstetric trauma that results in injury to the anal sphincter muscles and loss of muscle volume in old age [59]. New therapies are focusing on regeneration of the smooth and skeletal muscles within the sphincter to restore continence using tissue engineering strategies [60]. One of the first in vivo studies targeting faecal incontinence using biomaterials was published in 2013 by Kang et al. Myoblasts loaded onto PCL beads were injected into a canine model of sphincter injury. Unfortunately, the study did not produce any evidence of functional or histological improvement. However, the use of myoblasts in combination with biomaterials is advantageous, as myoblasts are particularly responsive to biomaterials. Their adhesion, differentiation and proliferation can be affected by surface topography, microstructure and mechanical properties [61, 62]. This effect could perhaps be manipulated to achieve more cell proliferation or have higher control of differentiation.

Shi et al. (2014) injected adipose-derived mesenchymal stem cells (AdMSCs) attached to silk fibrin microspheres into a model of urinary incontinence in rats. They found that there was long-term improvement (12 weeks) in the group administered cells attached to microspheres compared to the controls, as well as an improvement in leak pressure and decrease in lumen size [63]. These methods could potentially be applied to a human sphincter defect, specifically in the rhabdosphincter, which is thinner than the external anal sphincter [64]. For larger muscles, a porous scaffold may be more appropriate as they would promote vascularisation of the tissue, which is vital in regenerating healthy tissue.

### Tracheal tissue engineering

There are many challenges in tracheal tissue engineering. Clinically, tracheal stenosis, tracheomalacia, trauma due to prolonged intubation and cancers can all result in irreversible damage to the trachea [65] with resection of the trachea being the only treatment option available (up to half in an adult and a third in children) [66]. Therefore, there is a clear need for a suitable substitute of native tracheal tissue. A successful tracheal replacement would have to be biocompatible, have the rigidity to prevent collapse, but allow the flexibility usually provided by the native trachea. Once produced, the construct must also be able to vascularise and regenerate ciliated epithelium to become epithelialised [67]. Many different cell types, seeding techniques and materials are studied for this approach.

The most common cell types investigated for tracheal tissue engineering are chondrocytes and stem cells. The native trachea consists of cartilage rings, hence, why chondrocytes are often used as they produce collagen and aggrecan. Stem cells such as mesenchymal stem cells (MSCs), embryonic stem cells (ES) and induced pluripotent stem cells (iPS) have the ability to differentiate into many different cell lineages but, for this instance, are all used for epithelial cell regeneration. The cells are cultured with the scaffold to achieve re-epithelialisation similar to the native trachea.

Decellularised scaffolds are often considered for tracheal tissue engineering as they contain materials that form the ECM such as collagen, fibrin and hyaluronic acid [68], thus allowing cell adhesion, proliferation and differentiation [69, 70]. However, natural materials lack the mechanical integrity that synthetic materials provide [9, 71]. Thus, there are numerous studies combining natural materials, such as hydrogels with synthetic materials for tracheal tissue engineering to create a scaffold with the benefits of both natural and synthetic materials. Lin et al. (2011) combined PCL with a gelatin hydrogel to produce a tracheal scaffold which in an in vivo model showed suppression of the formation of scar tissue and increased survival compared to the PCL scaffold alone [72]. Growth factors can also be incorporated into hydrogel materials to allow for their sustained release over time within artificial trachea constructs. Tatekawa et al. (2010) loaded gelatin hydrogels with bFGF. For this purpose, bFGF could promote chondrogenesis and neovascularisation. A PLGA and collagen mesh was implanted around a defect in the tracheal rings of a rabbit; the scaffold was reinforced with a biodegradable stent, and the bFGF-loaded gelatin sheet was wrapped around the entire construct. The results showed that including bFGF into the scaffold resulted in improved cartilage regeneration within the tracheal defect and re-epithelialisation was observed after 6 months. It was reported that the construct had good mechanical strength; this is likely due to the implanted stent that would also prevent collapse [73]. Decellularised tracheal scaffolds have been used to replace the trachea of patients in various clinical trials [8]. A cadaver trachea is decellularised using a harsh detergent-enzymatic treatment that strips the extracellular matrix of any cells. The ECM scaffold is then re-seeded in a bioreactor, often with the patient’s own cells [74]. This process allows for cells to effectively attach and proliferate in a natural environment, and such a transplant eliminates the need for immunosuppressive therapy. Unfortunately, the decellularising procedure can damage the ECM; residual cells may remain in the scaffold, and the whole process requires a donor organ that is not always available. These constructs can often collapse, resulting in the need for recurrent implantation of stents to keep the airway open. The insertion of stents has been shown to lead to scarring and negatively affect re-epithelisation [8]. Preventing the collapse of this hollow organ has been at the forefront of research in the recent years. Gonzalez-Molina et al. (2012) has shown that using magnets to aid seeding of cells derived from connective tissues results in a significantly higher cell attachment to the scaffold compared to the use of established rotating/dynamic seeding methods [9, 75]. Park et al. (2015) designed a poly(l-lactide-co-ε-caprolactone) (PLCL) and gelatin scaffold that was rigid and flexible. The scaffold was seeded with chondrocytes and functionalised with transforming growth factor beta 1 (TGF-β1) growth factor to further stimulate regeneration of the cartilage. The cellularised scaffold was implanted, and in vivo studies showed that regeneration of the cartilage similar to native cartilage had been achieved and the organ did not collapse under compression [76]. 3D printing has also been used to produce scaffolds, Goldstein et al. (2015) printed polylactic acid grafts into a collagen gel. Once seeded with chondrocytes and implanted in vivo, the trachea became epithelialised and a new cartilage was produced [77]. Engineering the trachea has been successfully translated to the clinic [9]. Based on the outcome of these early studies, ongoing research addresses the challenges that have been observed in order to achieve an engineered construct with properties similar to the native tissue (epithelialised surface, cartilage rings, mechanical integrity to prevent collapse) [67].

## Conclusions

Tissue engineering of hollow organ constructs is no longer in its infancy. Advancements in engineering allows for a better understanding of the behaviour of biomaterials and their fabrication in the context of hollow organ construction. Biomaterials play an important role in underpinning many of the tissue engineering technologies being developed for hollow organs. Selection of appropriate biomaterials with suitable physicochemical properties is imperative to the success of hollow organ scaffold structures. The fabrication processes available are continuing to evolve to allow the shape, size, porosity, topography and scalability of the scaffold to be controlled in order to meet the tissue requirements [10]. Tissue engineering is a widely studied field with many research groups moving on from initial in vitro experiments to in vivo work, to further understand the effect and impact of their respective therapies in a more physiologically relevant environment, as well as highlighting the advancements in current research. With successes in recent clinical trials [7, 9], there is a promise to effectively replace other hollow organs. Despite this, there are still challenges that need to be addressed for successful engineering of hollow organs. This will include achieving complete vascularisation of constructs to ensure long-term survival in vivo. The success of engineering hollow organs lies in effectively bringing material engineering and biological principles together to address unmet clinical needs. This interdisciplinary field is rapidly developing and advancing, making for an exciting future for tissue engineering and regenerative medicine.

## Abbreviations

AdMSCs: adipose-derived mesenchymal stem cells
bFGF: basic fibroblast growth factor
CEHD: co-axial electrohydrodynamic processing
ECM: extracellular matrix
EHD: electrohydrodynamic
ES: embryonic stem cells
FGF: fibroblast growth factor
GI: gastrointestinal
IBD: inflammatory bowel disease
iPS: induced pluripotent stem cells
MSCs: mesenchymal stem cells
PBS: phosphate-buffered saline
PCL: polycaprolactone
PEG: polyethylene glycol
PET: polyethylene terephthalate
PGA: polyglycolic acid
PLCL: poly(l-lactide-co-ε-caprolactone)
PLGA: poly(lactic-co-glycolic acid)
PLLA: poly-l-lactic acid
TIPS: thermally induced phase separation
TGF-β1: transforming growth factor beta 1
UC: ulcerative colitis
VEGF: vascular endothelial growth factor
3D: three dimensional
